# A quantitative three-dimensional comparative study of alveolar bone changes and apical root resorption between clear aligners and fixed orthodontic appliances

**DOI:** 10.1186/s40510-023-00458-3

**Published:** 2023-02-27

**Authors:** Ibtehal Almagrami, Abeer A. Almashraqi, Bushra Sufyan Almaqrami, Amin S. Mohamed, Khaled Wafaie, Maher Al-Balaa, Yiqiang Qiao

**Affiliations:** 1 Department of Orthodontics, Faculty of Dentistry, First Affiliated Hospital of Zhengzhou University, No.1 Jianshe East Road Erqi District, Zhengzhou, Henan China; 2 Department of Orthodontics and Dentofacial Orthopedics, Faculty of Dentistry, Sana’a University, Sanaa, Republic of Yemen; 3 Department of Pre-Clinical Oral Health Sciences, College of Dental Medicine, QU Health, Qatar University, Doha, Qatar; 4 Department of Orthodontics, Hubei-MOST KLOS and KLOBM, School and Hospital of Stomatology, Wuhan University, Wuhan, China; 5 Ningbo Dental Hospital, Ningbo, Zhejiang China; 6 Department of Orthodontics, Xi’an Jiaotong Universit, Xi’an, China

**Keywords:** Alveolar bone thickness, Root resorption, Clear aligners, Fixed orthodontic appliances, CBCT

## Abstract

**Background:**

This study aimed to evaluate and compare the alveolar bone changes and to investigate the prevalence and severity of orthodontically induced inflammatory root resorption (OIIRR) of maxillary incisors in patients who received treatment with clear aligners (CA) versus conventional fixed appliances (FA), using cone-beam computed tomography (CBCT).

**Methods:**

One hundred sixty maxillary incisors from 40 patients with similar baseline characteristics based on the American Board of Orthodontics discrepancy index scores were divided into the CA and FA groups. The dentoalveolar quantitative changes were analyzed using pre- (T0) and post-treatment (T1) CBCT. The measured parameters included alveolar bone thickness (ABT), alveolar bone height (ABH), root length (OIIRR), and maxillary incisor inclinations.

**Results:**

Post-treatment, the average palatal and total ABT significantly decreased in central and lateral incisors in the FA group. In contrast, the CA group’s average labial ABT of the lateral incisors decreased considerably. Regarding the ABH, both groups showed significant labial and palatal marginal bone resorption. In both groups, root lengths significantly decreased after treatment (*p* < 0.005). The inter-group comparison revealed that ABT and root length had significantly decreased in the FA group compared to the CA group, while the ABH showed no significant difference between the two groups. The mean absolute reductions of ABT and OIIRR in the CA group were significantly less (− 0.01 ± 0.89 and 0.31 ± 0.42) than those in the FA group (0.20 ± 0.82 and 0.68 ± 0.97), respectively.

**Conclusions:**

CA and FA treatments appear to cause a significant ABT reduction and a statistically significant increased OIIRR in the maxillary incisor region, with a greater extent expected with FA treatment. However, the increased OIIRR values in the majority of both groups’ cases were not clinically significant. Both treatment modalities resulted in a significant ABH reduction, with the highest found in the labial side of lateral incisors in the CA group.

**Supplementary Information:**

The online version contains supplementary material available at 10.1186/s40510-023-00458-3.

## Introduction

The capabilities of fixed orthodontic appliance (FA) treatment paved the way to making it the most popular orthodontic appliance [1]. However, acceptance by the patients is often hindered by the appliance’s appearance and the patient’s ability to maintain proper oral health [2–4]. The rapid transformation in the dental sector hastens the shift toward patient-centered approaches and makes it mandatory to introduce new orthodontic appliances that meet both treating physician and patient needs. Clear aligner (CA) therapy has recently been introduced in orthodontics as a more esthetic and comfortable alternative to FA. The Align Technology 3D planning software “ClinCheck” enables practitioners to digitally plan treatment and preview all teeth movements up to the final result. Recently, CA has been used to treat a wide range of orthodontic cases, such as mild or moderate crowding, deep overbite, distalization, closing or opening of space, rotation, arch expansion, and others [5]. Nonetheless, its accuracy in clinical practice and compatibility with the designed movements on the software is still debatable in the literature [6]. Despite the limitations of both appliances, previous literature has shown that both appliances could be effectively used to treat mild and moderate crowding cases [7].

The concept of orthodontic tooth movement (OTM) in terms of both applications is based on bone remodeling theory and is mediated by the action of osteoclasts and osteoblasts through bone resorption and formation. The process, also known as “anabolic and catabolic therapeutic actions of the bone,” starts with the catabolism of bone on one side followed by anabolism on the opposite side of the intended-to-move tooth [8]. Some factors affect the degree of bone remodeling, such as the amount and intensity of the applied force, type of OTM (, i.e., controlled tipping, uncontrolled tipping, and transitional movement), alveolar bone morphology, and systemic condition or medications [9, 10]. Therefore, the proper understanding of physiological changes accompanied by OTM will prevent any iatrogenic sequelae in the periodontium, such as dehiscence and fenestration [11].

Another common iatrogenic effect of OTM that may jeopardize treatment success and tooth longevity is apical root resorption (ARR), which is defined as a physiological or pathological process that results in the shortening of the root apex [12, 13]. Orthodontically induced inflammatory root resorption (OIIRR) occurs in almost all cases, and its extent varies from one tooth to another [14]. Previous literature has shown an increased tendency for resorption in maxillary incisors and second premolars due to extensive tooth movement [15–17]. In addition to type and degree of OTM, other factors, such as genetics, previous trauma, age, nutrition, certain features of malocclusion, extraction/non-extraction treatment, type of appliance, type of applied force, and duration of treatment, may contribute to root resorption [12, 18]. Although many investigators have studied the prevalence and severity of OIIRR in orthodontic patients, it is still a complicated process that is not fully understood [19, 20].

According to a recently published umbrella review, it was found that there is an increase in OIIRR in maxillary incisors with FA therapy [21]. In comparison with CA, a recent systematic review by Gandhi et al. reported a non-significant difference between the two systems except for the maxillary right lateral incisors that showed less root resorption with CA [22], while other studies found that CA has a lower prevalence and severity of root resorption than FA [23–25]. These results should be interpreted with caution, as there was a difference in treatment duration, used method of detection (periapical, CBCT, cephalometric radiographs, orthopantomogram, and/or microscopic examination), and mechanism of action [7].

Diagnostic accuracy differs according to the used method of detection [26]. Previous studies reported that conventional two-dimensional (2D) radiography has inherent limitations due to magnification and distortion in contrast to CBCT, which helps to quantitatively assess alveolar bone and root length with high accuracy and precision [27], besides being highly reproducible [28] and exhibiting excellent accuracy and sensitivity [29]. Nonetheless, when using CBCT, special precautions should be taken, such as selecting a limited field of view CBCT and using protective devices to ensure patient safety [30].

Consequently, the primary aim of this retrospective study was to evaluate the dimensional alveolar bone changes that accompany OTM and to investigate the prevalence and severity of OIIRR in maxillary incisors within comparable groups treated with CA and FA. In addition, the secondary aim of this study was to compare the post-treatment alveolar bone changes and root resorption in maxillary incisors on either technique.

## Material and methods

This retrospective comparative study was approved by the Ethical Committee of the first affiliated hospital of Zhengzhou University, China, with reference number (2022-KY-0800-001).

The sample size was determined utilizing G*power 3.0.10 software with an alpha value of 0.05 and a power of 85% based on the study conducted by Li Yu et al. [23], who reported maxillary canine root resorption of 0.14 ± 0.53 and 1.53 ± 1.92 mm in the CA and FA groups, respectively. Power analysis showed a minimum sample of 18 subjects. However, the sample size was increased to 20 patients for each study group.

Orthodontic patients’ records were screened from January 1, 2018, to April 30, 2022. Patients were treated at the first affiliated hospital of Zhengzhou University, China, by a single experienced operator using either conventional FA or CA.

The inclusion criteria consisted of (1) adult patients aged ≥ 18 years, (2) mild to moderate crowding, (3) non-extraction treatment, (4) full permanent dentition (excluding third molars), and (5) good quality pre- (T0) and post-treatment (T1) records (CBCT, photographs, and model casts), which were obtained as part of their orthodontic diagnosis and treatment plan. Exclusion criteria included: (1) patients with previous maxillary incisor root canal treatment or history of trauma, (2) previous early interceptive or comprehensive orthodontic treatment, (3) history of maxillofacial trauma, (4) congenital anomalies (such as maxillary hypoplasia, cleft lip and/or palate), (5) systemic diseases, (6) smoker, (7) loss of periodontal attachment, (8) and/or evidence of prior inflammatory root resorption.

Based on the comprehensive periodontal examination done before orthodontic treatment that was reported thoroughly in the periodontal examination section of the patients’ files, any reported active inflammatory conditions showing deficient supportive periodontal tissues (osseous, soft tissue, or a combination of both types) were excluded. Only patients who were periodontally healthy before treatment were included in this study.

The American Board of Orthodontics (ABO) discrepancy index (DI) was used to evaluate the case difficulty in each group [31]. The ABO discrepancy index was used to measure the cases’ complexity in each group to maximize the comparability of the two studied groups, so cases with a high complexity score were excluded.

A total of 40 patients who met the inclusion criteria were divided into two equal groups (CA and FA). The CA group included 20 patients treated with Invisalign® (Align Technology, California, USA). The FA group included 20 patients treated with a fixed orthodontic appliance (Victory Series; 3 M Unitek®, California, USA).

Three-dimensional images were captured using a CBCT scan (KaVo® Dental GmbH, Bismarckring, Germany). The pre-treatment imaging parameters were 120 kV, 5 mA, 0.3 mm voxel size, and a field of view of 230 × 170 mm, which were identical to the post-operative imaging parameters. The patients were sitting upright with their teeth close to their maximum intercuspation. The Frankfort horizontal plane was positioned parallel to the floor, and the midsagittal plane was perpendicular to the floor. The collected CBCT scan data were transferred into a Digital Imaging and Communication in Medicine (DICOM) file format and then imported into the Invivo® 6 software program (Anatomage, San Jose, CA) to perform the 3D analysis.

**Fig. 1:**
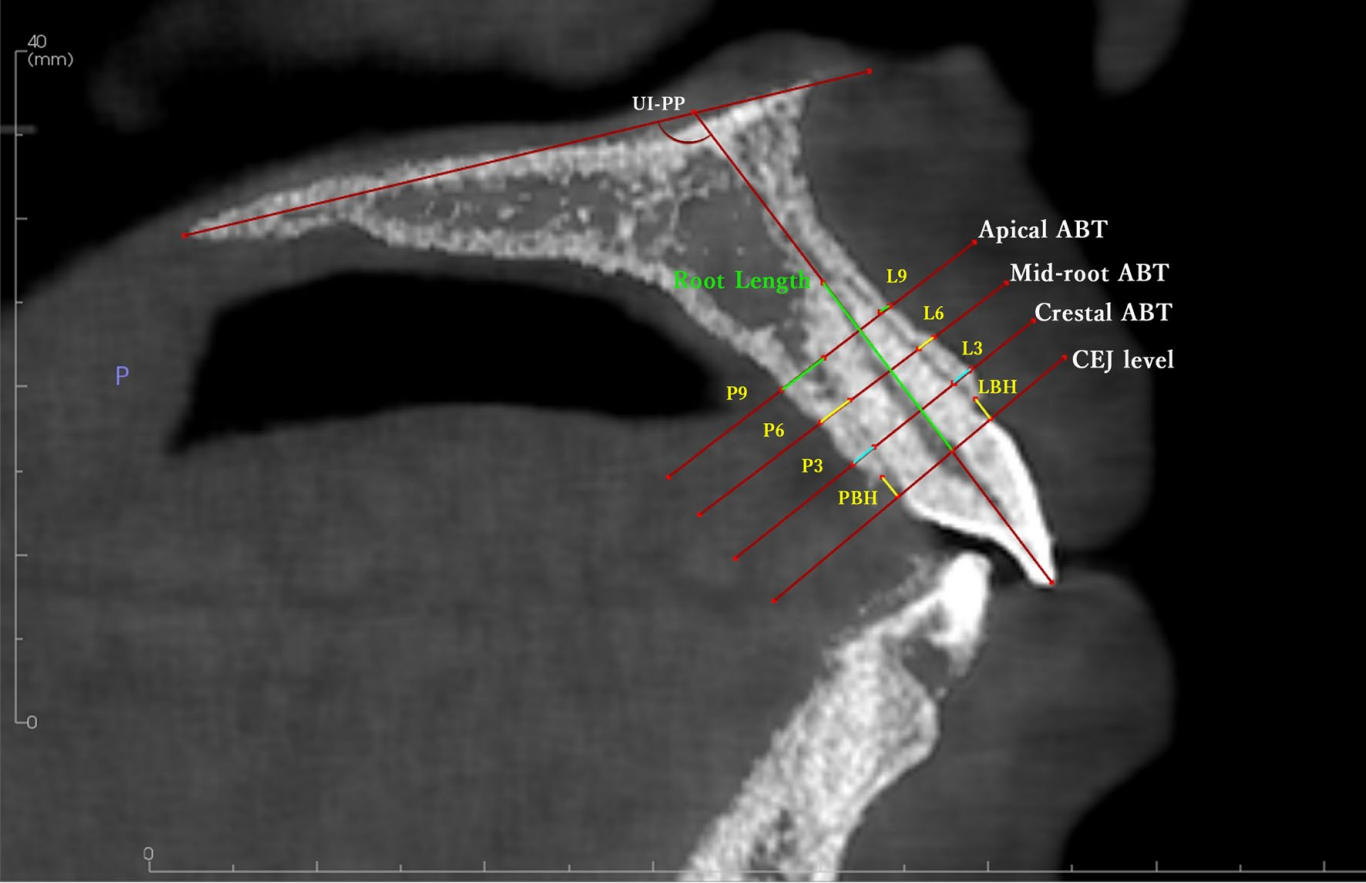
Reference lines and measurements in sagittal view for upper central incisor. ABT: alveolar bone thickness, LBH: labial bone height, PBH: palatal bone height, UI-PP: upper incisor to the palatal plane angle.

Axial, coronal, and sagittal planes of the CBCT volume were reoriented to be perpendicular to the long axis of each tooth under assessment. The tooth axis was defined as the line connecting the incisal midpoint to the center of the apical root foramen. Using the Invivo® 6 software, a reference line was drawn from the axial view, connecting the labial and palatal cementoenamel junction (CEJ). Labial and palatal alveolar bone thickness (ABT) was measured at three different levels with distances of 3, 6, and 9 mm from the CEJ in the apical direction, as seen in the axial view. The sections were referred to as the crestal, mid-root, and apical third (3, 6, and 9 mm, respectively). Labial and palatal alveolar bone height (ABH) was measured from the CEJ to the alveolar ridge crest. The distance between the constructed CEJ line and the root’s most apical point was measured to assess root length before and after treatment (Fig. 1). The inclination was measured as the angle between the upper incisor (UI) and the palatal plane (UI–PP).

Alveolar bone thicknesses, heights, root lengths, and the inclination of maxillary incisors on all pre-treatment CBCT scans were measured. Two weeks later, post-treatment measurements were done without access to the pre-treatment measurements. All measurements have been done under the supervision and guidance of oral and maxillofacial radiologists.

Sharpe’s method was used to score the severity of root resorption as follows [32]:

0° = No ARR (0 mm).

1° = Slight blunting of the root apex (1–2 mm).

2° = Moderate root apex blunting up to one-fourth root length (2 mm-1/4 root length).

3° = Excessive blunting of the root apex beyond one-fourth of the length of the root (> 1/4 root length).

Study models and data were coded to ensure the blindness of the researcher to the treatment groups during measurements and data analysis. To evaluate the reliability of measurements, intra- and inter-observer reliability of the measurements was assessed randomly after three weeks on records of 10 patients using intra-class correlation with confidence intervals of 95%. The examiners were blinded to the former measurements.

## Statistical analysis

The SPSS software (version 26; IBM Corp, Armonk, NY, USA) was used for all statistical analyses. Descriptive statistics, including each variable’s mean and standard deviation (SD), were calculated and presented as such. Data were checked for normal distribution using the Shapiro–Wilk test. Intra-group comparisons between pre-and post-treatment measurements were performed using the paired sample t test and the Wilcoxon signed-rank test. For inter-group comparisons, the independent sample t test and the Mann–Whitney U test were used. For measuring and comparing the severity and prevalence of OIIR between the two groups, the paired t test and chi-square test were used.

## Results

A total of forty patients were divided into two equal groups (CA and FA). The CA group included 20 patients (mean age 25.15 ± 6.67 years); the age distribution in both studied groups is presented in Additional file 1. The FA group included 20 patients (mean age 22.33 ± 4.33 years). No significant differences in overall treatment duration, CA patients (25.85 ± 8 months) and FA patients (29.67 ± 7.71 months) were detected. The overall ABO discrepancy index score of the CA (12.69 ± 0.52) group and the FA group (14.38 ± 0.89), indicating the baseline difficulty of the two groups, was similar (*p* = 0.610). No significant differences in the subjects’ baseline characteristics, including age, treatment duration, skeletal, dentoalveolar, bone thickness, bone height, root length, and maxillary incisor inclination between the two groups, were found as presented in Tables 1 and 2.

**Table 1:** Baseline characteristics and baseline discrepancy index (DI) of the two groups

	Fixed appliances (N = 20) Mean ± SD	Clear aligners (N = 20) Mean ± SD	*p* value
Age	22.33 ± 4.33	25.15 ± 6.67	0.128
Treatment duration	29.67 ± 7.71	25.85 ± 8.00	0.140
Overjet	0.75 ± 1.07	1.35 ± 1.31	0.121
Overbite	1.05 ± 1.10	1.20 ± 1.28	0.693
Anterior open bite	2.28 ± 3.55	1.00 ± 1.52	0.148
Lateral open bite	2.80 ± 4.42	1.25 ± 1.74	0.158
Crowding	2.25 ± 1.52	1.90 ± 1.17	0.418
Occlusion	2.10 ± 1.77	1.40 ± 1.85	0.229
Posterior crossbite	0.60 ± 1.14	0.55 ± 0.94	0.881
ANB angle	0.25 ± 1.12	0.60 ± 1.88	0.478
SN-MP angle	0.90 ± 3.21	1.19 ± 2.68	0.757
IMPA angle	1.30 ± 2.41	2.25 ± 3.48	0.3.22
Total score	14.38 ± 0.89	12.69 ± 0.52	0.610

SD: standard deviation

**Table 2:** Baseline characteristics comparison (T0-T0) of the two studied groups

			Fixed appliances (Mean ± SD)	Clear aligners (Mean ± SD)	*p* value
*Bone thickness*					
Central	Labial	S1	0.87 ± 0.39	0.84 ± 0.36	0.488^b^
		S2	1.10 ± 0.40	0.89 ± 0.37	0.147^b^
		S3	1.07 ± 0.64	1.00 ± 0.31	0.806^b^
		Average	1.07 ± 0.50	0.94 ± 0.35	0.048^b^
	Palatal	S1	1.65 ± 0.68	1.51 ± 0.52	0.855^a^
		S2	3.25 ± 1.07	3.00 ± 1.14	0.154^b^
		S3	4.73 ± 1.69	4.64 ± 1.86	0.122^b^
		Average	3.23 ± 1.74	3.05 ± 1.82	0.224^b^
	Total		2.15 ± 1.68	1.99 ± 1.68	0.209^b^
Lateral	Labial	S1	0.79 ± 0.54	0.85 ± 0.53	0.340^b^
		S2	0.94 ± 0.48	0.84 ± 0.58	0.287^b^
		S3	0.81 ± 0.51	0.73 ± 0.65	0.306^b^
		Average	0.84 ± 0.51	0.80 ± 0.58	0.740^b^
	Palatal	S1	1.34 ± 0.54	1.23 ± 0.55	0.430^a^
		S2	2.38 ± 0.88	2.37 ± 1.12	0.513^b^
		S3	3.97 ± 1.76	3.76 ± 1.34	0.468^b^
		Average	2.51 ± 1.38	2.45 ± 1.48	0.712^b^
	Total		1.67 ± 1.33	1.63 ± 1.39	0.787^b^
*Bone height*					
Central incisors	Labial		2.02 ± 0.59	2.10 ± 0.55	0.397^b^
	Palatal		1.47 ± 0.66	1.60 ± 0.51	0.083^b^
Lateral incisors	Labial		2.56 ± 1.43	2.84 ± 1.81	0.634^b^
	Palatal		1.71 ± 0.60	1.99 ± 1.53	0.473^b^
*Root Resorption*					
Central incisors			11.82 ± 1.68	12.14 ± 1.67	0.268^b^
Lateral incisors			12.50 ± 1.36	12.72 ± 1.24	0.356^b^
*Inclination*					
UI-PP			118.24 ± 6.65	117.63 ± 7.10	0.783^a^

a: Independent sample t test, b: Mann–Whitney U test

Intra- and inter-rater reliability for the repeated measurements was found to be non-significant (0.97 and 0.92, respectively).

### Intra-group comparison

Intra-group comparison results are reported in Table 3.

**Table 3:** Intra-group comparison (T0-T1) of the two studied groups

			Fixed appliances			Clear aligners		
			Pretreatment (Mean ± SD)	Post-treatment (Mean ± SD)	*p* value	Pretreatment (Mean ± SD)	Post-treatment (Mean ± SD)	*p* value
*Bone thickness*								
Central	Labial	S1	0.87 ± 0.39	0.70 ± 0.48	0.001^a^**	0.84 ± 0.36	0.70 ± 0.55	0.088^b^
		S2	1.10 ± 0.40	1.15 ± 0.58	0.839^b^	0.89 ± 0.37	0.95 ± 0.44	0.545^b^
		S3	1.07 ± 0.64	1.27 ± 1.00	0.407^a^	1.00 ± 0.31	0.91 ± 0.63	0.539^a^
		Average	1.07 ± 0.50	1.09 ± 0.74	0.205^b^	0.94 ± 0.35	0.85 ± 0.55	0.096^b^
	Palatal	S1	1.65 ± 0.68	1.31 ± 0.74	0.001^a^**	1.51 ± 0.52	1.24 ± 0.70	0.048^b^*
		S2	3.25 ± 1.07	2.69 ± 1.25	0.000^b^***	3.00 ± 1.14	3.05 ± 1.13	0.361^a^
		S3	4.73 ± 1.69	4.22 ± 2.06	0.022^b^*	4.64 ± 1.86	4.81 ± 1.58	0.125^b^
		Average	3.23 ± 1.74	2.76 ± 1.87	0.000^b^***	3.05 ± 1.82	3.03 ± 1.88	0.623^b^
	Total		2.15 ± 1.68	1.92 ± 1.64	0.000^b^***	1.99 ± 1.68	1.94 ± 1.76	0.596^b^
Lateral	Labial	S1	0.79 ± 0.54	0.45 ± 0.59	0.000^a^***	0.85 ± 0.53	0.62 ± 0.58	0.007**
		S2	0.94 ± 0.48	1.01 ± 0.61	0.607^b^	0.84 ± 0.58	0.65 ± 0.66	0.013^a^*
		S3	0.81 ± 0.51	0.93 ± 0.82	0.143^a^	0.73 ± 0.65	0.61 ± 0.72	0.068^a^
		Average	0.84 ± 0.51	0.80 ± 0.72	0.218^b^	0.80 ± 0.58	0.63 ± 0.65	0.000^b^***
	Palatal	S1	1.34 ± 0.54	1.12 ± 0.79	0.049^b^*	1.23 ± 0.55	1.24 ± 0.86	0.514^a^
		S2	2.38 ± 0.88	2.05 ± 1.22	0.022^a^*	2.37 ± 1.12	2.48 ± 1.29	0.037^b^*
		S3	3.97 ± 1.29	3.39 ± 1.76	0.036^a^*	3.76 ± 1.34	3.88 ± 1.56	0.034^b^*
		Average	2.51 ± 1.38	2.19 ± 1.61	0.001^b^**	2.45 ± 1.48	2.78 ± 1.96	0.000^a^***
	Total		1.67 ± 1.33	1.49 ± 1.42	0.001^b^**	1.63 ± 1.39	1.71 ± 1.81	0.137^b^
*Bone height*								
Central incisors	Labial		2.02 ± 0.59	2.94 ± 1.50	0.000^b^***	2.10 ± 0.55	3.00 ± 2.35	0.000^b^**
	Palatal		1.47 ± 0.66	2.30 ± 1.78	0.000^b^***	1.60 ± 0.51	2.29 ± 1.57	0.000^a^***
Lateral incisors	Labial		2.56 ± 1.43	3.41 ± 1.69	0.000^b^***	2.84 ± 1.81	4.85 ± 3.88	0.000^b^***
	Palatal		1.71 ± 0.60	2.73 ± 1.92	0.000^a^***	1.99 ± 1.53	2.44 ± 1.21	0.002^b^**
*Root Resorption*								
Central incisors			11.82 ± 1.68	11.14 ± 2.10	0.000^a^***	12.14 ± 1.67	11.86 ± 1.60	0.000^a^***
Lateral incisors			12.50 ± 1.36	11.95 ± 1.24	0.000^a^***	12.72 ± 1.24	12.38 ± 1.37	0.000^a^***
*Inclination*								
UI-PP			118.24 ± 6.65	119.47 ± 7.38	0.502^a^	117.63 ± 7.0	113.12 ± 5.42	0.044^a^*

a: paired sample t test, b: Wilcoxon signed-rank test, *: *p* < 0.05, **:*p* < 0.01, ***:*p* < 0.001

#### Bone thickness and height

Comparing pre- and post-treatment bone thicknesses in both groups, it was found that in the FA group, average palatal and total bone thickness had significantly decreased in central and lateral incisors, while in the CA group, a significant reduction in alveolar bone thickness was found in the average labial surface of lateral incisors (*p* < 0.005). Regarding ABH, a significant increase between T0 and T1 in both groups’ labial and palatal sides of maxillary central and lateral incisors (*p* < 0.005) was found, indicating labial and palatal alveolar bone resorption.

#### Root resorption

In terms of the OIIRR, the mean root length comparison between T0 and T1 in both groups showed that post-treatment root length significantly reduced in both groups (*p* < 0.000).

#### Incisor inclination

Post-treatment inclination of the upper incisors related to the palatal plane (UI-PP) had significantly decreased in the CA group (117.63 ± 7.10° and 113.12 ± 5.42°, respectively) while slightly increased in the FA group (118.24 ± 6.65° and 119.47 ± 7.38°, respectively; *p* > 0.05).

### Inter-group comparisons

Post-treatment changes comparison (T0-T1) of ABT, ABH, OIIRR, and maxillary incisor inclinations between FA and CA are presented in Table 4.

**Table 4:** Inter-group comparison (T0-T1) of the two studied groups

		Fixed appliances (Mean ± SD)	Clear aligners (Mean ± SD)	*p* value
*Bone Thickness*				
Central incisors	Labial	− 0.03 ± 0.70	0.09 ± 0.49	0.714^b^
	Palatal	0.47 ± 0.99	0.02 ± 1.09	0.000^b^***
	Total	0.22 ± 0.89	0.05 ± 0.85	0.004^b^**
lateral incisors	Labial	0.05 ± 0.55	0.18 ± 0.43	0.095^b^
	Palatal	0.32 ± 0.90	− 0.33 ± 1.18	0.000^b^***
	Total	0.18 ± 0.76	− 0.08 ± 0.92	0.001^b^**
Total		0.20 ± 0.82	− 0.01 ± 0.89	0.000^b^***
*Bone height*				
Central incisors	Labial	0.92 ± 1.36	0.90 ± 2.33	0.160^b^
	Palatal	1.83 ± 1.65	0.69 ± 1.50	0.419^b^
Lateral incisors	Labial	0.85 ± 1.22	2.01 ± 3.21	0.504^b^
	Palatal	1.03 ± 1.97	0.45 ± 1.90	0.381^b^
*Root Resorption*				
Central incisors		0.68 ± 0.97	0.28 ± 0.26	0.016^b^*
Lateral incisors		0.55 ± 0.54	0.35 ± 0.53	0.010^b^*
Average		0.62 ± 0.78	0.31 ± 0.42	0.000^b^***
*Inclination*				
UI-PP		− 1.23 ± 8.07	4.52 ± 8.03	0.030^a^*

a: Independent sample t test, b: Mann–Whitney U test, *: *p* < 0.05, **:*p* < 0.01, ***:*p* < 0.001

#### Bone thickness and height

The statistical comparison of post-treatment ABT changes showed that mean palatal and total ABT significantly decreased in both central and lateral incisors in the FA group compared to the CA group (*p* < 0.005). The mean absolute reductions of ABT in the FA group were significantly more (0.31 ± 0.42 and − 0.01 ± 0.89) than that in the CA group. ABH post-treatment changes showed no significant difference between the two groups, indicating a possible marginal bone loss in both groups during orthodontic treatment. The highest marginal bone resorption was found in the labial side of lateral incisors in the CA group (2.01 ± 3.21 mm).

#### Root resorption

OIIRR was significantly less in the CA group (0.31 ± 0.42 mm) than that in the FA group (0.62 ± 0.78 mm; *p* < 0.05). The highest mean root length reduction was found in central incisors (0.68 ± 0.97 mm) in the FA group.

The prevalence and severity of external root resorption were calculated to compare the OIIRR between the two groups. The prevalence in FA and CA were 83% and 69%, respectively, Table 5. Regarding the severity of OIIRR, in the CA group, 31.1% of the teeth showed 0° ARR, 67.50% showed 1°, and 1.3% showed 2°, while, in the FA group, 17.50% of the teeth showed 0° ARR, 80% showed 1°, 1.3% showed 2°, and 1.3% showed 3°. These results indicate that the OIIRR in the CA group was generally lower than that in the fixed appliances group, as shown in Table 6.

**Table 5:** Prevalence of root resorption in the two groups

	Fixed appliances %	Clear aligners %
Central incisors	80%	70%
lateral incisors	85%	67%
Average	83%	69%

**Table 6:** Classification of overall severity of apical root resorption in the two groups

Severity of AR	Fixed appliances (*n*, %)	Clear aligners (*n*, %)
0	14, 17.50%	25, 31.1%
1	64, 80%	54, 67.5%
2	1, 1.30%	1, 1.30%
3	1, 1.30%	0

#### Incisor inclination

**Fig. 2:**
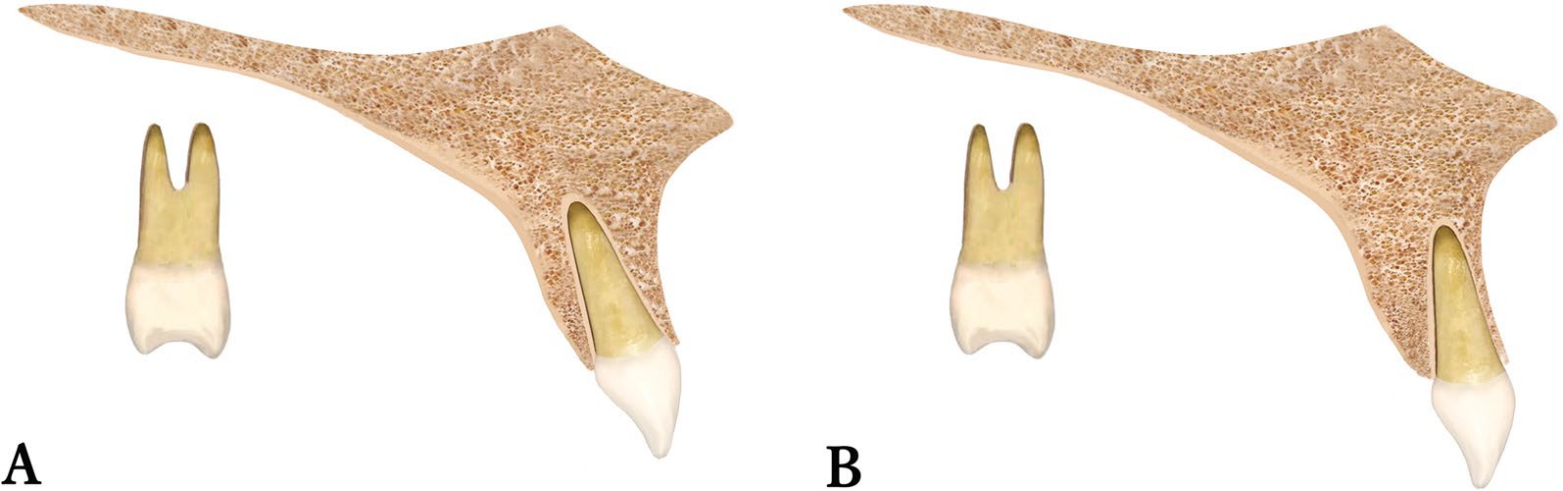
Estimated post-treatment inclination of upper incisors in fixed appliance group (**A**) and clear aligners group (**B**)

Regarding the comparison of the inter-group maxillary incisor inclination, a significant mean change between the FA and CA groups ( − 1.23 ± 8.07 and 4.52 ± 8.03, respectively), as a result of the significant decrease in incisor inclination in the CA group Fig. 2.

## Discussion

The finding of the current study shows that both treatment modalities resulted in increased labiolingual alveolar bone thickness reduction, marginal alveolar bone resorption, and external root resorption after treatment. FA group demonstrated a greater extent of labiolingual alveolar bone thickness reduction and root resorption, while the CA group demonstrated a significant decrease in labiolingual inclination.

Both groups had the same baseline characteristics and duration of treatment; furthermore, both groups were treated on a non-extraction base with mild to moderate crowding. Thus, relief of crowding was achieved by posterior teeth distalization, dental arch expansion, and proclination of anterior teeth. However, such treatment protocol for borderline cases could lead to excessive tooth movement, which may result in iatrogenic sequelae, such as root resorption, gingival recession, and alveolar bone loss. Hence, orthodontists have to thoroughly assess ABT and tooth position within the alveolar bone to improve the treatment outcome and avoid complications.

Due to the fact that CA treatment is often indicated for simpler cases with minor to moderate crowding and shorter treatment duration and due to its removable nature and light force, CA complications are assumed to be less when compared with FA treatment [22]. For that reason, the ABO discrepancy index (DI) and the baseline characteristics assessment in this study were carried out to ensure that patients treated with each technique had a comparable level of difficulty.

Intra-group analysis of T0-T1 changes showed a significant decrease in total ABT in the FA group due to the significant decrease in both labial bone thickness at the crestal level and palatal bone thickness at the three levels. Our findings were consistent with previous studies in the literature. Yodthong et al. reported that ABT changes were related to the change in incisor inclination caused by different types of root movement during orthodontic treatment [33]. According to their findings, labial crestal bone thickness and total ABT significantly decreased with the increase in incisor inclination caused by the torque movement of the maxillary incisors, while the apical bone thickness increased with the decrease in incisor inclination caused by the incisors’ tipping movements.

By contrast, intra-group analysis of T0-T1 changes in the CA group showed an increase in palatal mid-root and apical bone thickness, which resulted in an increase in the average palatal ABT and a decrease in the average labial bone thickness. This finding most likely occurred because of the significant decrease in incisor inclination caused by the aligners’ tipping movement of the maxillary incisors. Previous studies reported that clear aligners moved teeth by a tilting motion; however, controlling root movement with incisor labiolingual inclination is the major challenge for clear aligners, which coincides with the result of this study, which shows an increase in retroclination of incisors after treatment. They also found that when incisors were designed for lingual controlled tipping, pure tipping occurred for 36.1% of the incisors, which resulted in the labial movement of the roots and an increased risk of fenestration; therefore, additional lingual root torque might be needed to avoid severe labial bone defects [34].

In terms of the inter-group comparison, current study findings showed a significant palatal and total ABT reduction in both central and lateral incisors in the FA group when compared with the CA group. The study results suggest that the forces exerted by the FA group were heavier because of its nature as bodily movement with more control over root torque, while in the CA group, the applied forces were much less, which occurred as a result of the tipping movement.

Regarding ABH changes after orthodontic treatment with both FA and CA, this study’s findings showed that labial and palatal ABH significantly increased in both groups. No statistically significant difference was found in the inter-group comparisons, indicating marginal bone level was compromised in both groups. These findings match the results of previous studies [35, 36]. The highest marginal bone resorption in this study was found in the labial side of lateral incisors in the CA group (2.01 ± 3.21 mm). Increased alveolar bone height above 2 mm is termed bone dehiscence. According to Sheng et al., post-treatment maxillary alveolar bone dehiscence increased by 19% in patients treated with fixed appliances [35]. Zhang et al. [36] found in their recent study that the mandibular labial and lingual alveolar bone resorption in CA patients was 0.78 and 0.34 mm, respectively, indicating that the labial alveolar bone loss was greater than lingual alveolar bone loss, which is consistence with our study findings. In this study, the labial and lingual alveolar bone loss in the CA group was 1.64 and 0.57 mm, respectively, while it was 0.89 and 1.43 mm, respectively, in the FA group. The possibility of a spontaneous repair of the alveolar bone defect caused by orthodontic treatment is still unknown. Lee et al. [37] found that if the post-orthodontic treatment marginal bone resorption was less than 1 mm, regeneration of bone to its original condition is possible. Therefore, preventing the occurrence or aggravation of alveolar bone resorption is crucial.

Previous studies have reported a correlation between the development of alveolar bone defects and alveolar bone thickness on the corresponding side and a correlation with the type of tooth movement and inclination [33]. For example, continuous tooth movement in the case of maxillary incisors retraction at the point at which the tooth apex is near or in contact with the bone cortex (thin labial bone) might result in the creation of an alveolar bone defect if the remodeling rate of the alveolar bone is slower than the rate of tooth movement. Therefore, it is essential to ensure that the root apex remains within the alveolar bone during orthodontic movement of the maxillary incisors; if not, labial bone fenestration and lingual alveolar bone resorption might occur during retraction of the incisors. Hence, prior to starting the treatment, the incisor position within the alveolar bone must be determined, and the tooth movement type must be carefully planned.

The inclination changes and the anteroposterior root apex movement are factors associated with labial and lingual marginal alveolar bone resorption. When the root apex moves closer to the cortical bone, greater alveolar bone loss on the same side tends to occur [36]. In our study, the root apex of the maxillary incisors moved more labially in the CA group, and average tooth inclination decreased significantly, which was associated with more labial marginal bone resorption. While in the fixed appliance group, the root apex moved lingually and average tooth inclination slightly increased, resulting in more lingual marginal bone resorption.

Concerning OIIRR, the intra-group comparison showed significant root resorption in central and lateral incisors in both groups (*p* < 0.000). At the same time, inter-group comparisons showed that the prevalence and severity of OIIRR in the CA group (68.50% and 0.31 ± 0.42 mm, respectively) were significantly less than those in the FA group (82.50% and 0.62 ± 0.54 mm, respectively). Previous studies reported mean OIIRR for comprehensive treatment with FA ranging from 1.36 to 1.42 mm [16, 17, 38]. A study used periapical radiographs found that the mean external root resorption of maxillary incisors treated with FA was 2.26 mm [39]. Another study used CBCT found that patients who received FA had mean resorption in maxillary incisors of 0.59 mm [40]. In our findings following FA, maxillary central and lateral mean root resorption values were 0.62 mm. Regarding CA, 2 mm maxillary incisor ARR was reported on the periapical, panoramic, and cephalometric radiographs [41]. While using CBCT, another study reported a mean value of maxillary incisor root resorption of 0.51 mm [42], and another study found that maxillary incisors length was shortened by 0.13 mm [23]. Our study found that after CAT, the mean value of maxillary incisor root resorption was 0.31 mm. The conflicting results concerning the outcome of OIIRR might be due to the difference in the selection of imaging tools, the magnitude of applied force, sample size, and the type of selected appliance.

Compared with FAs, previous studies have reported that CAs are associated with a lower risk of OIIRR due to the applied light and intermittent forces programmed in the aligners [24, 25]. FAs apply heavier forces than CAs and control the labiolingual inclination of the incisor by controlling the incisor root torque, a significant predictor for external root resorption [43]. Therefore, the probability of root healing by encouraging the cementum repair process is greater with CAT than with FAT. Additionally, treatment with CAs is subject to individual compliance, in which noncompliance leads to more intermittent force delivery and a shorter duration of force application, resulting in less root resorption [25].

CBCT imaging provides a great diagnostic and assessment modality for alveolar bone dimensional changes and root resorption. A study compared buccal bone height and thickness measurements on both CBCT and direct measurements on cadavers found strong agreement [27]. Another study found a simple non-significant risk of fenestration and dehiscence overestimation on CBCT [44]. However, this finding has no conflict with our study since it reminds us that even if the situation is not so bad, we must remain cautious. Generally, external root resorption is hard to detect with 2D radiographs when the root resorption is minimal or resorption at one aspect (mesial, distal, buccal, or mid-apical) [45]. Furthermore, root resorption values were found to be overestimated and less accurate with 2D than with CBCT [22, 45]. It has been reported that panoramic radiography may overestimate the prevalence of ARR by 20% compared with periapical radiography [16]. Moreover, according to Gandhi et al. [22] CBCT shows a decreased magnitude of ARR than 2D radiographs, and that is why 2D radiographs may overestimate the amount of ARR with orthodontic treatment. Also, it was reported by Deng et al. [45] that the ARR value of CBCT was lower than the 2D as a result of magnification and distortion in 2D images.

One of the limitations of this study was the sample size; although calculated in advance and the resulting sample size was used, the main challenge that prevented increasing the sample size was equating the baseline characteristics of the two groups based on the ABO DI and matching of pre-treatment measurements. Hence, future studies with a larger sample size may provide more information on the dimensional changes of alveolar bone and root resorption during orthodontic treatment with FAs and CAs. Another limitation of this study is that data analysis was carried out on immediate post-treatment CBCT images; therefore, we cannot state whether or not the orthodontic treatment sequel of both treatment modalities undergoes spontaneous reparative changes over time unless a comprehensive long-term assessment is done; this is recommended for future similar research. Although the 3D identification of the apical root area is accurate and valid [8, 26, 46, 47], volumetric measurements using manual segmentation might give a better presentation of root resorption and are recommended in any further research.

## Conclusion

Within the limitations of this study, it was concluded that

- FA treatment causes a significant decrease in alveolar bone thickness of both maxillary incisors, particularly on the palatal side, while CA treatment resulted in a significant labial bone thickness reduction of the maxillary lateral incisors.
- Treatment with FA and CA causes significant labial and lingual marginal alveolar bone loss around maxillary incisors, with the highest found in the labial side of lateral incisors in the CA group.
- FA and CA treatments resulted in statistically significant increased OIIRR in the maxillary incisor region with higher prevalence and severity of OIIRR in patients receiving FAs. However, the increased OIIRR values in most of both groups’ cases were not clinically significant.
- The post-treatment buccolingual inclination of maxillary incisors in the CA group significantly decreased due to labial root movement, indicating a compromised capability of CA to achieve adequate palatal root movement; such an issue may increase the possibility of labial bone fenestration.

Finally, different types of tooth movement using different appliance designs and orthodontic biomechanics might have different side effects than those reported in this study.

## Supplementary Information

**Additional file 1.** The age distribution in fixed appliances (FA) and clear aligner (CA) groups.

## Data Availability

Data used and/or analyzed during the current study are available from the corresponding author on reasonable request.

## Abbreviations

OIIRR: Orthodontically induced inflammatory root resorption
CA: Clear aligners
FA: Fixed appliances
CBCT: Cone-beam computed tomography
ABO: American Board of Orthodontics
ABT: Alveolar bone thickness
ABH: Alveolar bone height
OTM: Orthodontic tooth movement
ARR: Apical root resorption
2D: Two-dimensional
DI: Discrepancy index
CEJ: Cementoenamel junction
UI: Upper incisor
UI-PP: Palatal plane
