# Supplementary material for: A quantitative three-dimensional comparative study of alveolar bone changes and apical root resorption between clear aligners and fixed orthodontic appliances
Source: Prog Orthod. 2023 Feb 27;24:6. doi: 10.1186/s40510-023-00458-3 (PMC9968667; doi:10.1186/s40510-023-00458-3)
Supplement: Supplementary file 1 — Additional file 1. The age distribution in fixed appliances (FA) and clear aligner (CA) groups. [file 40510_2023_458_MOESM1_ESM.docx]

Supplementary material 1: The age distribution in fixed appliances (FA) and clear aligner (CA) groups
